# Rectum Perforation Secondary to Ingested Chicken Bone Presenting as Peri-Anal Abscess: A Case Report

**DOI:** 10.7759/cureus.49673

**Published:** 2023-11-29

**Authors:** Feras ALsannaa, Reem Bin Saleem, Jawaher ALowayyid

**Affiliations:** 1 Trauma and Acute Care Surgery, Prince Sultan Military Medical City, Riyadh, SAU; 2 General Surgery, Prince Sultan Military Medical City, Riyadh, SAU

**Keywords:** horseshoe abscess, peri-anal abscess, fishbone, ingested foreign body, rectal perforation

## Abstract

Rectal perforation secondary to an ingested foreign body is a rare occurrence that can be challenging to diagnose. It may initially present as a perianal abscess. Herein, we report a rare incident involving a patient who presented with a perianal abscess. The initial assessment and an abdominal CT scan revealed a large horseshoe perianal abscess with a small linear hyperdensity noted near the anal verge. The patient was taken to the operating room, where he was found to have perforated the rectum due to an ingested chicken bone. The procedure involved the incision and drainage of the abscess, along with the removal of the foreign body.

## Introduction

Perianal abscess is one of the most common surgical conditions in general surgery that presents to the emergency department. It is usually caused by an infection of the glandular crypts in the rectum or anus [1]. Rarely an ingested foreign body can pass through the entire gastrointestinal tract and become impacted in the anorectal area, causing injury to the anorectal mucosa and perforation, which can lead to subsequent infection and abscess formation. Although rare, perianal abscess can be caused by rectal perforation secondary to an ingested foreign body, as reported in a few cases in the literature [2-4]. We are reporting a rare case of rectal perforation secondary to an ingested chicken bone in a gentleman who presented with a perianal abscess.

## Case presentation

A 42-year-old male with morbid obesity presented to our emergency department complaining of perianal pain and swelling for one week, associated with subjective fever, chills, diarrhea, and pus discharge from the anus. The patient denied a history of nausea, vomiting, abdominal pain, or trauma. The patient reported that he presented to the emergency department six days prior with the same complaint; but was managed conservatively as a case of hemorrhoid. His surgical history was significant for a hemorrhoidectomy one year prior.

On a physical exam in the emergency department, he was febrile with a temperature of 39.4°. However, the remaining vital signs were within the normal range. His abdomen was soft, without any tenderness, guarding, or rigidity. Examination of the perianal area revealed induration and tenderness around the anus. The digital rectal examination could not be completed due to severe pain. However, the presence of pus discharge from the anus was noted. Laboratory evaluation was significant for a neutrophil-predominant leukocytosis of 19,24 × 109/L.

**Figure 1:**
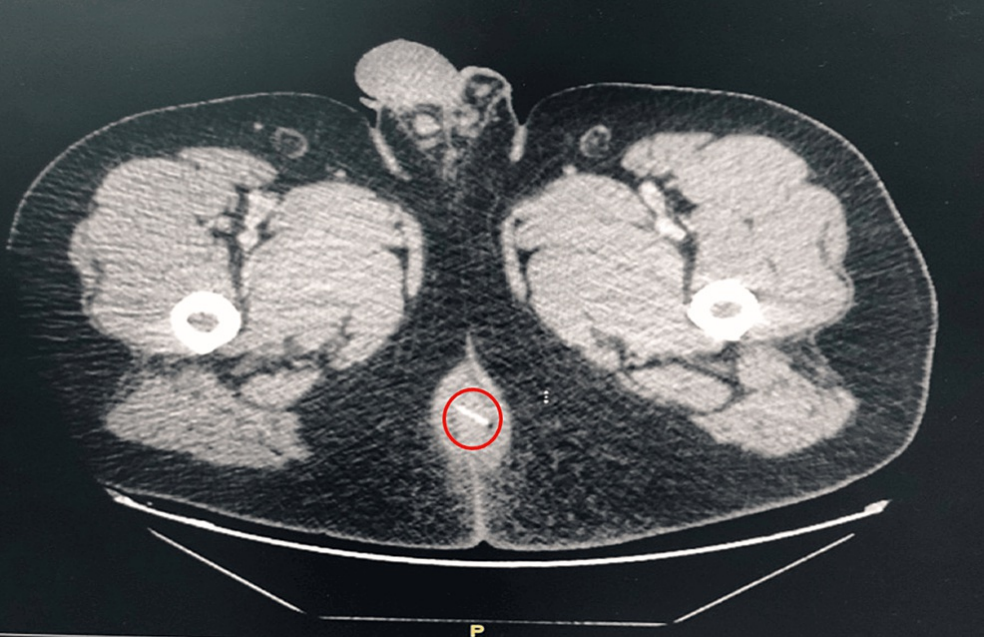
A CT scan of the pelvis (axial section) shows the linear foreign body (red cycle).

A contrast-enhanced computed tomography of the abdomen and pelvis (CT) revealed a large horseshoe perianal abscess. It measured approximately 3.0 ×5.4×7.7 cm and had evidence of left external sphincter breach and extension into the left ischioanal fossa, with superior extension along the left pelvic floor muscle, left ischiorectal extension, and left-sided supralevator extension. A linear hyperdensity was noted close to the anal verge (Figure 1).

**Figure 2:**
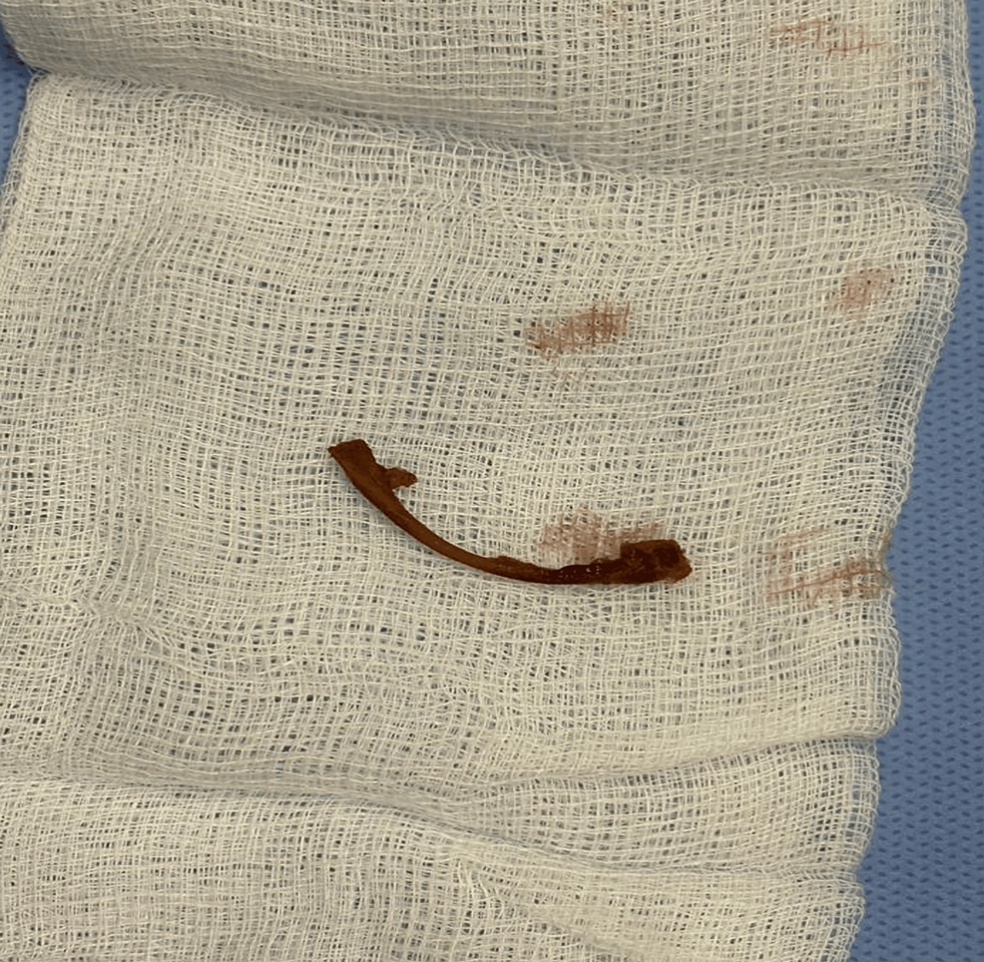
Intraoperative photo of the rectal foreign object.

The patient was initially started on broad spectrum antibiotics, then he was taken to the operating room for anorectal examination under anesthesia and an incision and drainage of the perianal abscess. Under general anesthesia, with the patient in lithotomy position, the examination revealed a rectal perforation localized at the 3 o'clock position, about 6 cm from the anal verge, transrectal drainage was performed, resulting in a large amount of purulent drainage, and a chicken bone was removed from the abscess cavity (Figure 2), the wound cavity was then thoroughly irrigated with saline, and a foleys catheter was inserted in the cavity for drainage. The patient tolerated the procedure well and was then transferred to the ward, where he continued intravenous antibiotics and received daily dressing.

**Figure 3:**
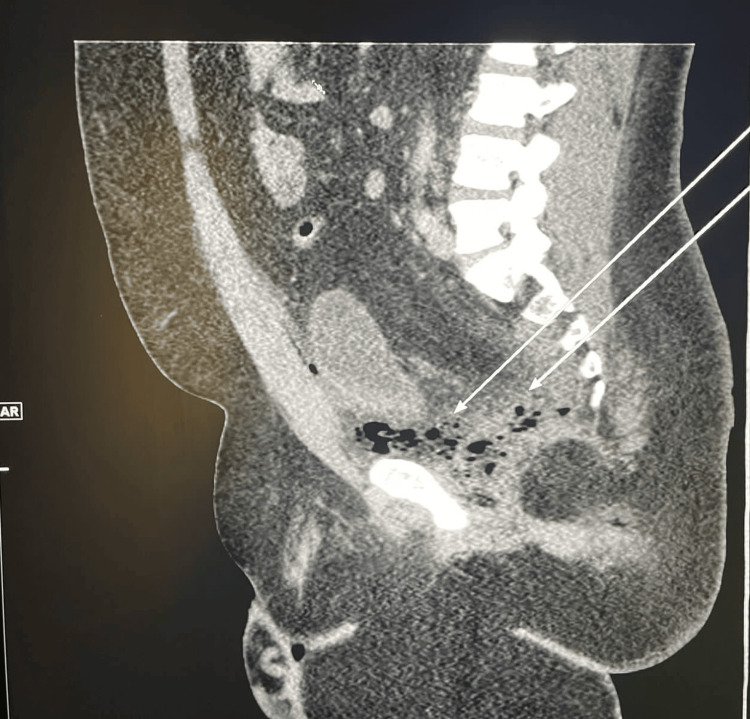
CT scan of the pelvis demonstrating the abscess collection (white arrows).

However, three days after the initial procedure, the patient started complaining of urinary incontinence. A CT scan was obtained, which showed a new development of left pelvic extraperitoneal collection with mottled gaseous Foci extending anterior to the urinary bladder. It measured 10.3×6.4 (Figure 3). The patient was taken to the operating room, and an incision was made in the left gluteal area over the most fluctuant part. A large amount of purulent material was drained, and then the cavity was thoroughly irrigated and lightly packed. 

He was discharged home on post-operative day 10 and recovered appropriately. He has been following up as an outpatient in the clinic and is doing well without any subsequent complications.

## Discussion

Foreign body ingestion in adults is a common event that usually happens accidentally while ingesting food. The most commonly ingested foreign bodies in the adult population are organic materials, such as fish bones (9%-45%) and chicken bones (8%-40%). In about 80% of cases, the ingested foreign bodies pass through the gastrointestinal tract without causing any acute complications. However, in approximately 20% of the cases, the foreign body can get impacted and lead to life-threatening complications, including obstruction, perforation, fistula formation, hemorrhage, and collection [5].

Foreign body impaction typically occurs at anatomical points of angulation and narrowing, such as the esophageal sphincters, pyloris, duodenum, cecum, ileocecal valve, appendix, the sigmoid colon, and the anus. Gastrointestinal perforation resulting from foreign body ingestion is rare, with only 1% of ingested foreign bodies causing bowel perforation, with fish bone reported as the most common object causing perforation. The most common sites of perforation by foreign bodies are at the ileocecal valve and rectosigmoid junction. The risk of perforation depends on the length as well as the sharpness of the ingested foreign body.

Gastrointestinal perforation secondary to a foreign body can present a wide spectrum of clinical presentations, including acute or chronic abdominal pain, bowel obstruction, and gastrointestinal hemorrhage. In cases of rectal or anal perforation, as presented in our case, the patient can also present with a peri-anal abscess [6]. The length of time between ingestion and perforation varies widely and can range from hours to years; the mean reported time to perforation is 10.4 days. In rare instances, when a foreign body penetrates the bowel wall, it can migrate into adjacent visceral structures such as the abdominal wall, bladder, peritoneum, and liver, causing additional complications [7].

It can be challenging to diagnose because patients presenting with symptoms are usually nonspecific and mimic other surgical emergencies. A careful history and physical examination should be obtained. However, in the majority of cases, patients may not recall a history of foreign body ingestion, so anorectal perforation secondary to a foreign body is rarely diagnosed pre-operatively.

Laboratory investigation can show leukocytosis and an increase in inflammatory markers. Plain radiograph images of the abdomen and chest should be obtained first in all suspected cases. A study conducted by Ngan and colleagues showed the X-ray sensitivity of ingested fish and chicken bones, which are usually radiodense enough, is 32% with a specificity of 91%. However, in some cases, X-rays can be inconclusive as the foreign body may be concealed by soft tissue mass or fluids, especially in cases of low perforation, such as low rectum and anus. In these cases, a computed tomography (CT) scan is more informative, with a sensitivity of 100% and a specificity of 91%, as reported by Coulier et al. [8,9].

The management of foreign body ingestion depends on the stability of the patient, the presenting symptoms, the nature of the ingested foreign body, and the location of the foreign body at diagnosis. Broad-spectrum antibiotics should be initiated in all cases of suspected perforation [10]. The type of surgical intervention depends on the location of the perforation, the stability of the patient, the time of presentation, and the degree of contamination. Adequate drainage of the perianal abscess caused by a perforated foreign body and ensuring the removal of the ingested foreign body are key in managing such cases.

## Conclusions

Rectal perforation caused by an ingested chicken bone is a rare occurrence. It can present as a perianal abscess and should be considered by physicians. Early recognition and surgical intervention, including incision and drainage of the abscess with removal of the foreign body, are essential for successful treatment.
